# Splenic Flexure Colon Cancer Presenting as a Perisplenic Abscess With Subsequent Intrasplenic Abscess Formation and Portal Venous Gas: A Case Report

**DOI:** 10.7759/cureus.107672

**Published:** 2026-04-24

**Authors:** Hiroyuki Hazama, Kazumasa Nakamura, Kohei Koido, Takeshi Oshima, Kou Ohata

**Affiliations:** 1 Gastrointestinal Surgery, Shizuoka General Hospital, Shizuoka, JPN

**Keywords:** contained perforation, elective resection, intrasplenic abscess, percutaneous drainage, perisplenic abscess, portal venous gas, splenic abscess, splenic flexure colon cancer

## Abstract

Splenic abscess associated with colon cancer is rare, and progression from a perisplenic abscess to an intrasplenic abscess is particularly unusual. We report the case of a 70-year-old man who presented in February 2023 with abdominal pain and fever. Contrast-enhanced computed tomography showed a perisplenic abscess and wall thickening at the splenic flexure. After percutaneous drainage and antibiotic therapy, an intrasplenic abscess and portal venous gas developed. Following infection control, colonoscopy and biopsy confirmed splenic flexure colon adenocarcinoma, and elective open left hemicolectomy with distal pancreatectomy and splenectomy was performed. Gross and histopathological examination showed no direct tumor invasion into the splenic parenchyma. This case is worth documenting because it illustrates an unusual pattern of splenic abscess progression in colon cancer and highlights the importance of considering an underlying colorectal malignancy in patients presenting with localized inflammatory abscesses around the spleen.

## Introduction

Splenic abscess is a rare infectious condition that may develop through a variety of mechanisms, including hematogenous spread, infective endocarditis, immunocompromised status, trauma, and contiguous spread of inflammation from adjacent organs [1]. In contrast, splenic abscess associated with colon cancer is extremely rare, and the literature is limited to a small number of case reports [2-5].

Proposed mechanisms of splenic abscess formation in colon cancer include direct tumor invasion, splenocolic fistula formation, and localized spread of infection associated with contained perforation or microperforation [2-5]. However, cases in which serial imaging suggests progression from a perisplenic abscess to an intrasplenic abscess in the absence of direct splenic invasion are rarely documented. Reporting such a case may improve recognition of atypical infectious presentations of splenic flexure colon cancer and may help guide diagnostic reassessment and treatment planning after initial infection control.

## Case presentation

A 70-year-old man presented in February 2023 with fever and abdominal pain. His medical history included duodenal ulcer, multiple cerebral infarctions with residual mild left hemiparesis and cognitive impairment, chronic hepatitis C after sustained virologic response, multiple gastric ulcers, hypertension, and dyslipidemia. He had been receiving warfarin for secondary prevention of cerebral infarction.

Approximately one month before presentation to our hospital, he developed abdominal pain and fever. He was treated with antibiotics at a previous hospital for suspected intra-abdominal infection; however, his inflammatory response showed insufficient improvement, and he was referred to our institution. He did not report any remarkable change in bowel habits, hematochezia, or melena. On arrival, his temperature was 36.5°C, and the other vital signs were stable. Physical examination revealed mild tenderness in the left flank without rebound tenderness or muscular guarding. Laboratory findings on admission are summarized in Table 1 and were notable for anemia, a marked inflammatory response, hypoalbuminemia, elevated hepatobiliary enzyme levels, and coagulation abnormalities attributable to warfarin therapy.

**Table 1:** Laboratory findings on admission

Parameter	Result	Unit	Reference range
White blood cell count	8.9	x 10^3^/μL	3.3-8.6
Hemoglobin	9.4	g/dL	13.7-16.8
Platelet count	270	x 10^3/^μL	158-348
C-reactive protein	17.18	mg/dL	≤0.14
Total protein	6.4	g/dL	6.6-8.1
Albumin	2.5	g/dL	4.1-5.1
Total bilirubin	1.7	mg/dL	0.4-1.5
Aspartate aminotransferase	154	U/L	13-30
Alanine aminotransferase	148	U/L	10-42
Alkaline phosphatase	133	U/L	38-113
Blood urea nitrogen	18	mg/dL	8-20
Creatinine	0.80	mg/dL	0.65-1.07
Sodium	138	mmol/L	138-145
Potassium	3.5	mmol/L	3.6-4.8
Chloride	106	mmol/L	101-108
Prothrombin time	68.4	s	-
Prothrombin time activity	7	%	70-130
International normalized ratio	5.88	-	-
Activated partial thromboplastin time	98.1	s	22.3-37.1
Carcinoembryonic antigen	2.0	ng/mL	≤5.0
Carbohydrate antigen 19-9	4.0	U/mL	≤37

After admission, warfarin was discontinued, and anticoagulation was switched to continuous intravenous unfractionated heparin.

**Figure 1:**
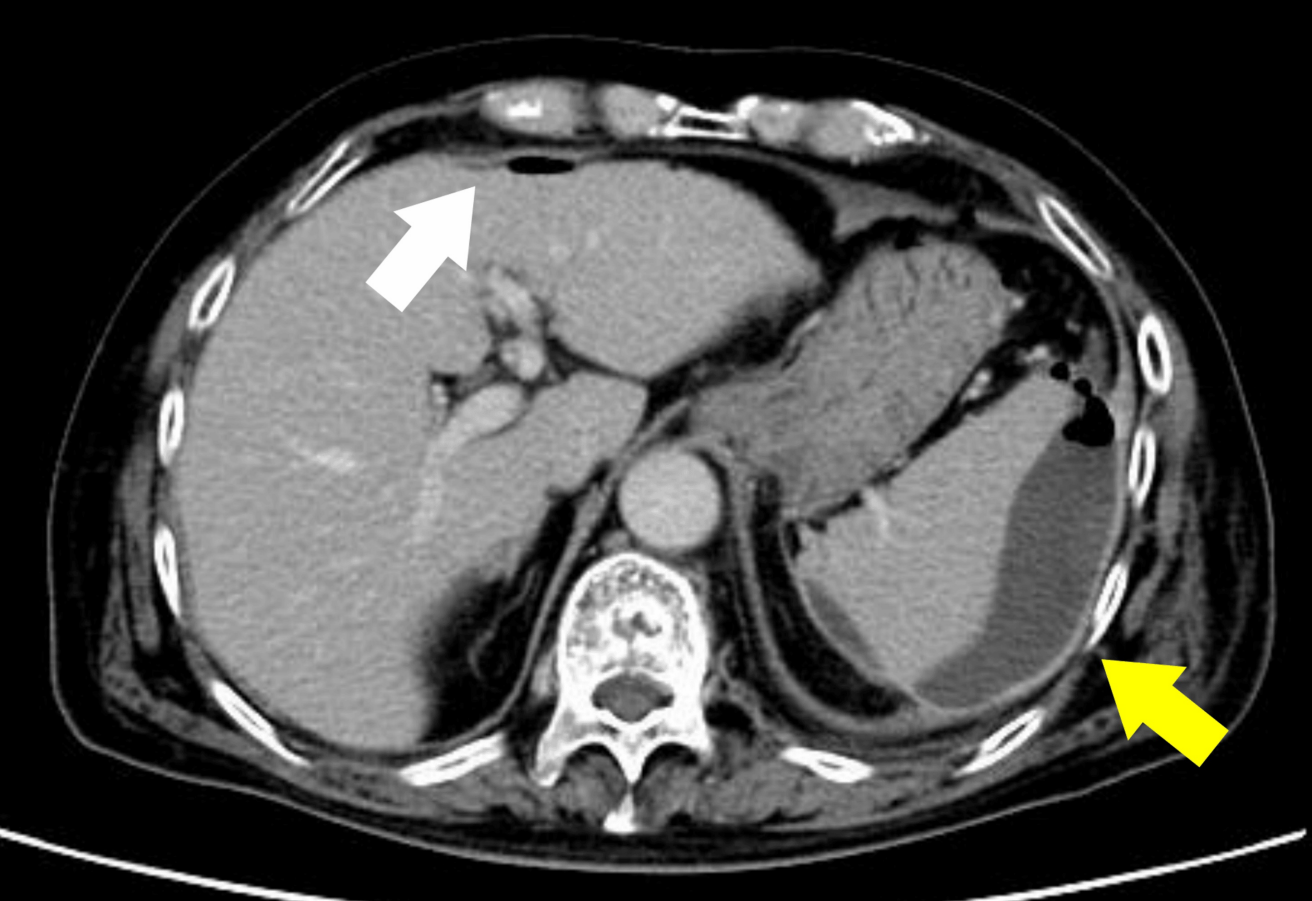
Contrast-enhanced CT at presentation showing a perisplenic abscess. An encapsulated fluid collection containing gas was seen extending from the perisplenic space to the left subphrenic space, consistent with a perisplenic abscess (yellow arrow). A small amount of free intraperitoneal air was also present along the liver surface (white arrow).

**Figure 2:**
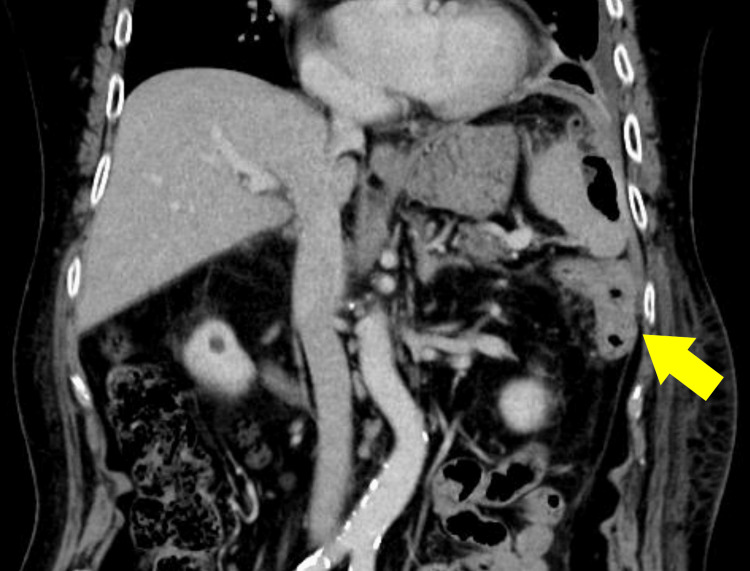
Contrast-enhanced CT at presentation showing wall thickening at the splenic flexure. Wall thickening with contrast enhancement was seen at the splenic flexure of the colon (yellow arrow).

Because the patient presented with an acute intra-abdominal inflammatory process, intravenous contrast-enhanced CT was selected for initial evaluation, and oral or rectal contrast was not considered essential for immediate management. Contrast-enhanced abdominal CT demonstrated an encapsulated fluid collection measuring up to 9.8 cm extending from the perisplenic space to the left subphrenic space, with a small amount of free intraperitoneal air around the collection and along the liver surface (Figure 1). Wall thickening with contrast enhancement was also seen at the splenic flexure of the colon (Figure 2).

Based on these findings, a perisplenic abscess secondary to contained perforation of splenic flexure colon cancer was suspected. Although perforated diverticulitis was also considered initially, colon cancer could not be excluded, and conservative treatment, including percutaneous drainage, was selected first.

**Figure 3:**
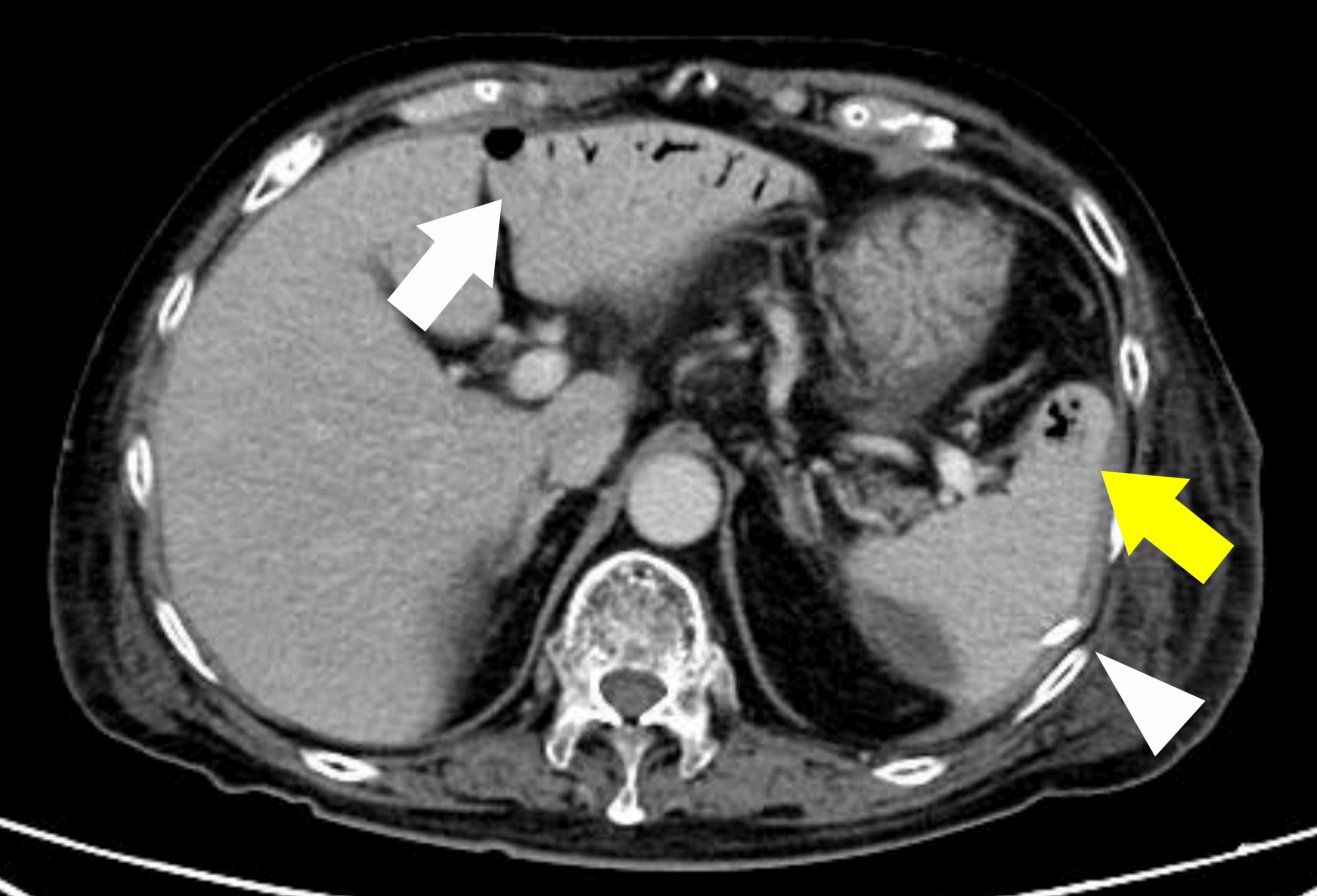
CT findings after percutaneous drainage on hospital day 3. After placement of a pigtail catheter, the perisplenic abscess had decreased in size (white arrowhead). In contrast, a new gas-containing abscess had appeared within the splenic parenchyma, consistent with an intrasplenic abscess (yellow arrow). Portal venous gas was also seen in the liver (white arrow).

On the day after admission, percutaneous drainage was performed through the left flank using a 7-Fr pigtail catheter. Feculent drainage was obtained, and a peripherally inserted central catheter was placed for nutritional management. Broad-spectrum antibiotics were continued and adjusted according to the culture results. On hospital day 3, CT showed shrinkage of the perisplenic abscess, but newly demonstrated portal venous gas in the liver and a gas-containing abscess within the splenic parenchyma (Figure 3).

However, the patient had no worsening abdominal pain or peritoneal signs, and CT showed no findings strongly suggestive of bowel ischemia. Although extension of the perisplenic infection into the splenic parenchyma was suspected, conservative management was continued with careful monitoring while keeping the possibility of occult bowel ischemia in mind.

**Figure 4:**
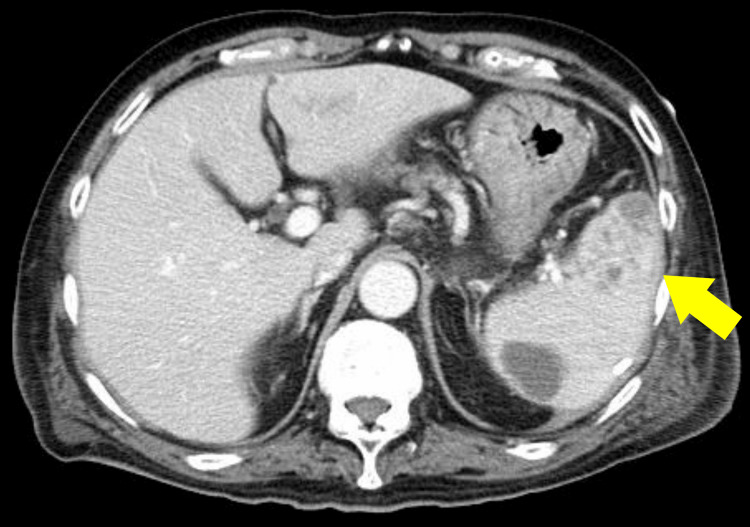
CT findings after percutaneous drainage on hospital day 9. The portal venous gas and free intraperitoneal air had disappeared, but the intrasplenic abscess had enlarged to approximately 6 cm in maximum diameter (yellow arrow).

Blood cultures obtained at admission grew Bacteroides fragilis, and cultures of the drainage fluid grew *Enterobacter* spp. and *Escherichia coli*, indicating severe infection with bacteremia. Thereafter, his general condition remained relatively stable, and the inflammatory markers gradually improved. On hospital day 9, CT showed the disappearance of the portal venous gas and free intraperitoneal air, although the intrasplenic abscess had increased in size (Figure 4).

**Figure 5:**
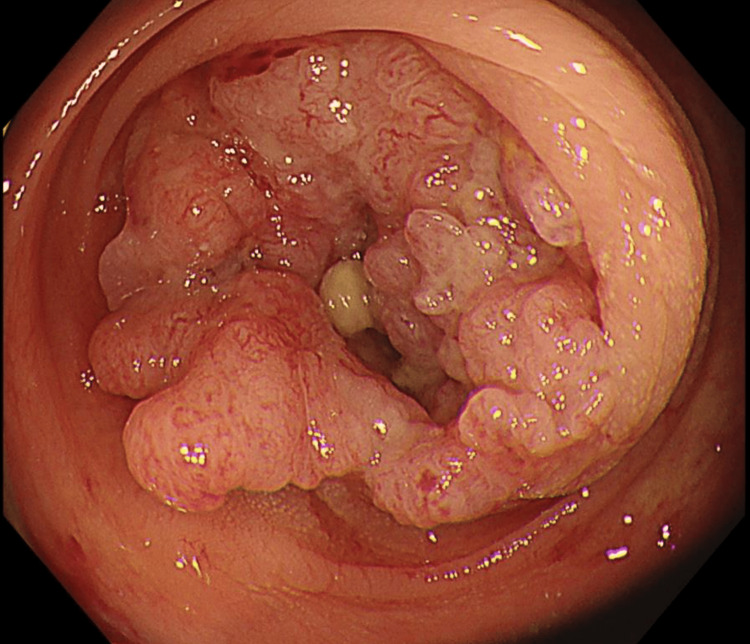
Colonoscopic findings on hospital day 20. A circumferential ulcerated lesion corresponding to a type 2 lesion in the Japanese classification was identified at the splenic flexure.

Because his abdominal findings and inflammatory response were improving, elective surgery was planned. On hospital day 16, CT showed slight shrinkage of the intrasplenic abscess, with further improvement in the inflammatory findings. After infection control and improvement in his general condition had been achieved, colonoscopy was performed on hospital day 20. It revealed a circumferential ulcerated lesion at the splenic flexure, corresponding to a type 2 lesion according to the Japanese Classification of Colorectal, Appendiceal, and Anal Carcinoma (Figure 5) [6].

The biopsy showed well-differentiated tubular adenocarcinoma, and splenic flexure colon cancer was diagnosed as the cause of the contained perforation and abscess formation. Elective surgery was performed on hospital day 31. The patient underwent open left hemicolectomy with distal pancreatectomy and splenectomy. Because the perisplenic abscess cavity was densely adherent to the abdominal wall, diaphragm, and colon, and because possible splenic invasion could not be excluded intraoperatively, en bloc resection including the abscess was performed. The operative time was 6 hours 29 minutes, and blood loss was 1,395 mL.

Postoperatively, the patient required transfusion for anemia, nasogastric decompression for postoperative ileus, management of aspiration-related chemical pneumonitis and sputum retention, and pancreatic stump drain management. Nevertheless, his overall condition gradually improved. The drain was removed on postoperative day 21, and he was discharged on postoperative day 25.

**Figure 6:**
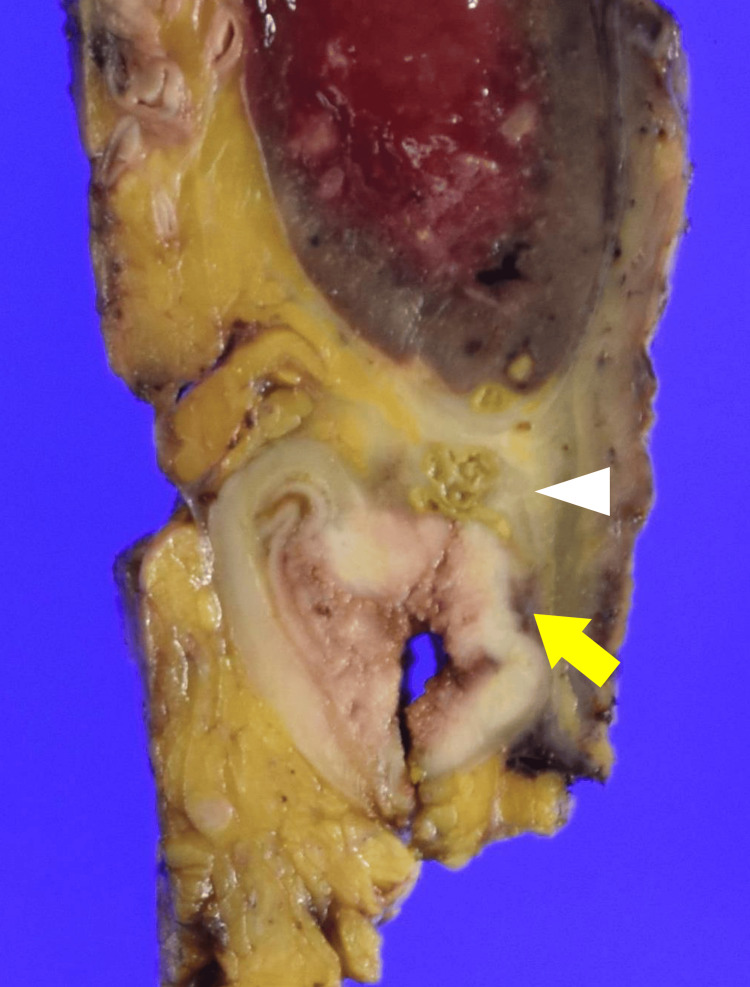
Gross findings of the resected specimen. The colonic tumor is indicated by the yellow arrow, and the abscess cavity by the white arrowhead. On the cut surface, no obvious direct invasion from the colonic tumor into the splenic parenchyma was identified.

Gross examination of the resected specimen showed a tumor at the splenic flexure and abscess formation in the spleen. On the cut surface, there was no obvious direct invasion from the colonic tumor into the splenic parenchyma (Figure 6).

**Figure 7:**
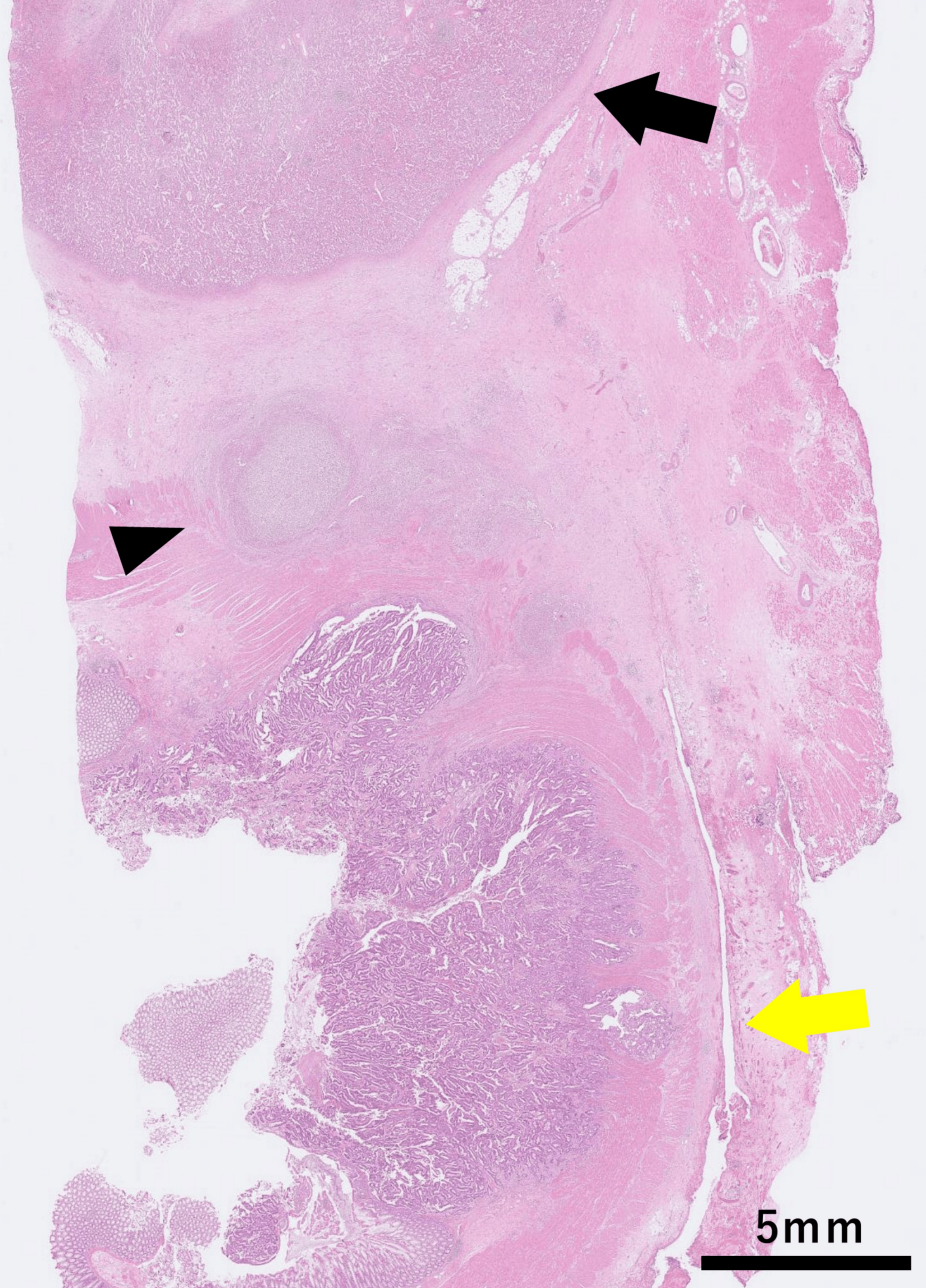
Histopathological findings of the resected specimen. Histopathological examination showed moderately differentiated tubular adenocarcinoma. The tumor invaded the subserosa without serosal penetration (yellow arrow). No direct invasion into the splenic parenchyma was identified (black arrow), and a small abscess was interposed between the tumor and the spleen (black arrowhead). Scale bar = 5 mm.

Histopathological examination revealed moderately differentiated tubular adenocarcinoma, pT3N0M0, pathological stage IIA according to the Union for International Cancer Control (UICC) TNM Classification of Malignant Tumours, 8th edition [7], with negative resection margins. The tumor invaded the subserosa without serosal penetration, and no direct invasion into the splenic parenchyma was identified. A small abscess was interposed between the tumor and the spleen (Figure 7).

Adjuvant chemotherapy was discussed with the patient, but was not administered after shared decision-making. At approximately 18 months after surgery, no obvious recurrence had been identified.

## Discussion

Splenic abscess is a rare infectious disease, reported in 0.14-0.7% of autopsy cases, and is known to arise from hematogenous spread, trauma, immunocompromised status, infective endocarditis, and contiguous inflammatory spread from adjacent organs [1]. Splenic abscess associated with colon cancer is extremely rare, with only a few reported cases in the literature [2-5].

The most important feature of the present case is that the splenic abscess most likely did not result from direct tumor invasion into the spleen. Rather, it appears to have developed first as a perisplenic abscess and then progressed into an intrasplenic abscess. Serial CT images showed that the perisplenic abscess preceded the appearance of a gas-containing low-density lesion within the splenic parenchyma. In contrast, gross examination of the resected specimen and histopathological analysis revealed no direct tumor invasion into the splenic parenchyma. Taken together, these findings suggest that the splenic abscess developed when a perisplenic abscess caused by contained perforation of the splenic flexure colon cancer extended from the splenic capsule into the splenic parenchyma. Previous reports have also suggested that, in addition to direct invasion, splenocolic fistula formation and localized infectious spread associated with contained perforation or microperforation may underlie splenic abscess formation in colon cancer [2-5]. Therefore, this case is noteworthy because it demonstrates that progression from a perisplenic abscess to an intrasplenic abscess can occur even in the absence of direct splenic invasion.

Another important finding in this case was the presence of portal venous gas during the course of splenic abscess formation. Portal venous gas has classically been regarded as a serious radiologic sign suggestive of bowel necrosis. However, with the widespread use of CT, it is now recognized in a variety of conditions, including intra-abdominal abscess, gastrointestinal inflammation, sepsis, and perforation [5,8]. In the present case, there was no worsening abdominal pain, no peritoneal signs, and no CT findings strongly suggestive of bowel ischemia. Therefore, the portal venous gas was considered more likely to have resulted from an infectious mechanism related to the splenic abscess than from bowel necrosis itself. Accordingly, the presence of portal venous gas alone should not automatically mandate emergency laparotomy; rather, the decision should be based on a comprehensive assessment of abdominal findings, overall clinical status, laboratory data, and serial contrast-enhanced CT findings to determine whether bowel ischemia or progressive peritonitis is present [5,8].

From the standpoint of treatment strategy, infection control was prioritized in this case by percutaneous drainage and antibiotic therapy, followed by elective curative resection. The World Society of Emergency Surgery (WSES) guidelines for colon and rectal cancer emergencies note that perforation at the tumor site may remain localized and that management should consider both sepsis control and oncologic resection [9]. In the present case, differentiation from inflammatory disease was difficult initially; however, once infection control had been achieved, colonoscopy allowed definitive identification of the primary lesion, and radical resection could then be performed. In cases initially presenting as localized inflammatory abscesses, perforated colorectal cancer should remain an important differential diagnosis. After control of sepsis and improvement of the acute inflammatory process, colonoscopic evaluation is important to identify the primary lesion and exclude underlying malignancy [9-11].

## Conclusions

Splenic abscess associated with splenic flexure colon cancer is rare. In the present case, the lesion did not involve direct splenic invasion by the tumor; instead, it initially presented as a perisplenic abscess, later progressed to an intrasplenic abscess, and was accompanied by portal venous gas. This case highlights the importance of considering an underlying colorectal malignancy in patients presenting with localized inflammatory abscesses. Even in the presence of portal venous gas, management should be based on the overall clinical and radiologic findings.
